# Barriers and Enablers to Delegating Malnutrition Care Activities to Dietitian Assistants

**DOI:** 10.3390/nu14051037

**Published:** 2022-02-28

**Authors:** Alita Rushton, Judith Bauer, Adrienne Young, Heather Keller, Jack Bell

**Affiliations:** 1 Department of Nutrition and Dietetics, The Prince Charles Hospital, Chermside, QLD 4032, Australia; alita.rushton@health.qld.gov.au; 2 School of Human Movement and Nutrition Sciences, The University of Queensland, St. Lucia, QLD 4072, Australia; j.bauer1@uq.edu.au; 3 Department of Nutrition, Dietetics & Food, School of Clinical Sciences, Monash University, Notting Hill, VIC 3168, Australia; 4 Royal Brisbane Women’s Hospital, Department of Nutrition and Dietetics, Herston, QLD 4029, Australia; adrienne.young@health.qld.gov.au; 5 Centre of Health Services Research, The University of Queensland, Herston, QLD 4029, Australia; 6 Schlegel-UW Research Institute for Aging, Waterloo, ON N2L 3G1, Canada; hkeller@uwaterloo.ca; 7 The Prince Charles Hospital, Allied Health, Chermside, QLD 4032, Australia

**Keywords:** assistants, allied health personnel, delegation, professional, delivery of healthcare, hospitals, malnutrition, nutritional support, qualitative research

## Abstract

Delegation of malnutrition care to dietitian assistants can positively influence patient, healthcare, and workforce outcomes. However, nutrition care for hospital inpatients with or at risk of malnutrition remains primarily individually delivered by dietitians—an approach that is not considered sustainable. This study aimed to identify barriers and enablers to delegating malnutrition care activities to dietitian assistants. This qualitative descriptive study was nested within a broader quality assurance activity to scale and spread systematised and interdisciplinary malnutrition models of care. Twenty-three individual semi-structured interviews were completed with nutrition and dietetic team members across seven hospitals. Inductive thematic analysis was undertaken, and barriers and enablers to delegation of malnutrition care to dietitian assistants were grouped into four themes: working with the human factors; balancing value and risk of delegation; creating competence, capability, and capacity; and recognizing contextual factors. This study highlights novel insights into barriers and enablers to delegating malnutrition care to dietitian assistants. Successful delegation to dietitian assistants requires the unique perspectives of humans as individuals and in their collective healthcare roles, moving from words to actions that value delegation; engaging in processes to improve competency, capability, and capacity of all; and being responsive to climate and contextual factors.

## 1. Introduction

Malnutrition can occur when patients are not meeting the increased nutrition requirements related to their condition or complications or when uptake of macronutrient intake is insufficient [1]. Although dependent on populations and diagnostic criteria applied, estimates suggest approximately one-third of inpatients are malnourished, contributing negatively to patient morbidity, physical and functional recovery from disease, injury, mortality, length of stay, and financial burden on the health system [2,3,4,5,6].

Nutrition care practices that are dependent on specialist delivery, for example, dietitians, medical nutrition specialists, or nurse practitioners, are not sustainable for all patients with or at risk of malnutrition. This is in part due to the time- and resource-intensive nature of individualised nutrition care, increased service demand, and funding allocations for specialist nutrition services [7,8,9,10]. Malnutrition screening is foundational to supporting timely nutrition care and directing resources towards those who are malnourished or at risk of malnutrition. Overarching international, national, and statewide guidelines and recommendations support screening on admission and periodically using a tool that is validated for the setting in which it is applied [11,12]. Although nutrition care practices vary from site to site and are also governed by local policies, procedures, and protocols, most sites in Queensland commonly use a malnutrition screening tool (MST) for malnutrition screening, and subjective global assessment (SGA) for malnutrition assessment and diagnosis, with recommendations for screening on admission and weekly screening thereafter [8,12,13].

Recent evidence demonstrates that 73% of patients at risk of malnutrition do not require specialised nutrition care from a dietitian but supportive nutrition care [14]. Supportive nutrition care is a category of nutrition care that provides opportunities for systematised, interdisciplinary interventions, for example, general high-protein, high-energy education. The inclusion of delegation to dietitian assistants to ensure efficient effective provision of supportive nutrition care has been a highlighted opportunity [8]. 

Healthcare reform is driving change to ensure high-value healthcare provision to consumers [15,16]. Consequently, systematised, interdisciplinary approaches, including delegation to the dietitian assistant workforce, are encouraged [8,9,14,17]. The assistant staff for the dietetic profession are also known as nutrition assistants, dietitian or dietetic assistants, dietary aides, dietary or dietetic support workers, diet or dietetic technicians, menu monitors, and/or allied health assistants, and their roles may vary; in this paper, we will refer to this role as “dietitian assistant”. In the Australian state where the research was conducted, there is no consistent requirement for qualification, titles, registration, or training to be employed as a dietitian assistant; unpublished data suggest at least some of the workforce have certificate-level (or higher) qualifications. Governance for delegation requires a balance across national, state, and local frameworks, policies, procedures, guidelines, and task instructions. Dietitian assistant scope of practice, role descriptions, and requirements vary across sites, both within Australia and globally. Literature is limited regarding the demographics, education, and training background of the dietitian assistant workforce, activities undertaken in the dietitian assistant role, and the models of care in which they are embedded. However, a recent systematic review concluded that patient, healthcare, and workforce outcomes appear to be positively influenced by the role of dietitian assistants across all elements of the nutrition care process [18]. Dietitians have completed either a four-year bachelor’s degree or a relevant bachelor degree with additional postgraduate training.

Knowledge and attitudes surrounding delegation appear positive, with dietitians and dietitian assistants reporting high confidence in delegation of malnutrition care activities [19]. However, a recent report of nutrition care practice, within Queensland hospitals in Australia, suggests poor uptake of delegated nutrition care processes, with 75% or more of dietitians continuing to individually undertake malnutrition care activities [19]. The dietetic profession has identified and prioritised delegation of elements of malnutrition care as opportunities to facilitate systematised interdisciplinary malnutrition care; however, it was suggested that further investigation into the complexities surrounding implementation of delegation to dietitian assistants is required [20]. Identifying barriers and enablers to delegation has been highlighted as a crucial step for translation and change in practice [19,20]. Consequently, the aim of this study is to use a qualitative approach to identify barriers and enablers to delegating malnutrition care activities to dietitian assistants from the perspectives of dietitian assistants, dietitians, and management.

## 2. Materials and Methods

This qualitative descriptive study was nested within a broader quality assurance activity to scale and spread systematised interdisciplinary malnutrition program for implementation and evaluation (SIMPLE) [8,14]. This study was timed to be undertaken during the early-implementation phase. Participants were recruited using a purposive sampling technique to maximize diversity across seven Queensland public hospitals (Table 1). Ethical approval for this study was received from the Prince Charles Hospital HREC (ID47929) and the University of Queensland (Number 2019000845).

### 2.1. Data Collection

Semi-structured interview questions were developed by two independent researchers. Question development was informed by individual behaviour change theory [21] and contextual factors [22]. The full list of interview questions can be found in Appendix A (Table A1). The primary question participants were asked was, “What are your thoughts around delegating malnutrition care activities to assistants?”. The interviews were conducted over a two-month period (February to March 2020) by the lead researcher for six of the seven sites. The interviews at the seventh site were conducted by another researcher, as the lead researcher works as a dietitian assistant at that site. 

Interviews were conducted face-to-face where possible, with one interview conducted via video conference (using Zoom^®^). Before commencing the interview, participants completed a demographic form and were provided a statement regarding interviewer bias and voluntary participation. A semi-structured approach was applied wherein participants were asked the primary question, following which the interviewer engaged the participant in conversation using the interview schedule as a guide, adding in additional questions for further explanation and exploration as required. In line with Laur et al. [23], interviews were conducted beyond saturation, as they formed a basis for a broader implementation of the SIMPLE program.

### 2.2. Data Analysis

Data analysis was undertaken using the inductive thematic analysis method by Braun and Clarke [24]. A summary of key interview findings was provided to management to inform SIMPLE implementation. Verbatim transcriptions were completed by the lead researcher using Microsoft Word^®^ and Express Scribe Transcription Software^®^. Data familiarisation occurred by listening to interview recordings and reviewing field notes, and handwritten notes for insights and reflections made during the transcription process. As per Saldana [25], first- and second-level coding was undertaken by the lead researcher; ideas within the data were initially identified, and all candidate themes were reviewed to produce provisional themes and sub-themes. Themes and sub-themes with accompanying descriptions and exemplar quotes were provided to two co-researchers, along with four uncoded transcripts for validation of emerging themes and cross checking; a random number generator was used to select these transcripts for review. Initial themes and sub-themes were amended following initial feedback. The updated document of the themes, sub-themes, descriptions, exemplar quotes, and visual representations was forwarded to additional co-researchers for further development of themes. Themes and sub-themes were refined based on this feedback. NVIVO^®^ software was used to support data analysis.

## 3. Results

Twenty-three interviews were conducted across seven sites and ranged from 13 to 40 min; site descriptions can be found in Appendix B (Table A2). There was similar representation of dietitian assistants (30%), dietitians (26%), and managers (30%), with slightly fewer food services staff participating (13%). Most participants were female, with dietitians and managers mostly working full time and dietitian assistants mostly working part time (Table 2). The majority of dietitians interviewed had ≤10 years of experience (83%); however, the experience of dietitian assistants was more diverse. Most of the participants from management and food services had >10 years of experience (Table 2).

**Figure 1:**
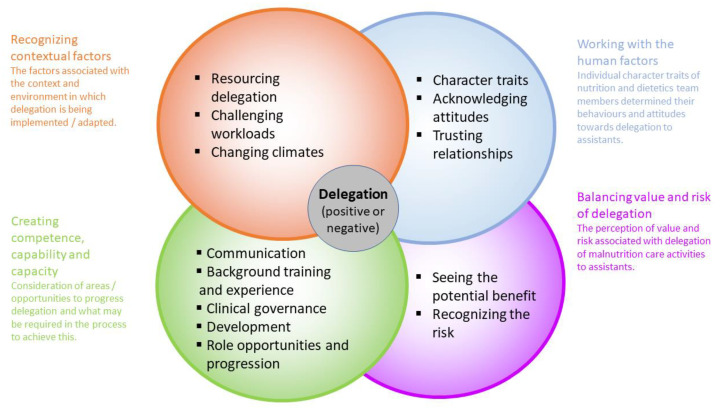
Visual representation of factors influencing delegation of malnutrition care to dietitian assistants.

Barriers and enablers to delegation of malnutrition care to dietitian assistants were grouped into four themes: working with human factors; balancing value and risk of delegation; creating competence, capability and capacity; and recognizing contextual factors. Figure 1 provides a visual representation of the themes and sub-themes and demonstrates interactions among themes.

### 3.1. Working with the ‘Human Factors’

Individual character traits, attitudes and beliefs, and trusting relationships influenced delegation of malnutrition care to dietitian assistants. Character traits determined nutrition and dietetics team members’ behaviours and attitudes towards delegation to dietitian assistants. Managers and dietitian assistants consistently highlighted character traits of dietitians perceived to adversely influence their decision to delegate and utilise their assistant staff. When discussing participants’ thoughts around delegation of malnutrition care to dietitian assistants, multiple respondents referred to the point that there is always a human element; “*So as much as I guess you try to make it umm a process, all of those human factors come into [it]*” (Management, Site B). Based on participants’ responses, the innate character and/or personality traits of staff affected delegation. These traits that enabled delegation included willingness, positivity, and trust. Lack of dietitian openness to change was also consistently reported; “*it could be that the dietitian inherently has a barrier to delegating and is looking for reasons why I can’t delegate*” (Food Service, Site A). Dietitian harm avoidance, fear, risk aversity, stubbornness, narrowmindedness, apathy, perfectionism, and distrust were also perceived as barriers to delegation. “*That umm almost like perfectionist mentality (mmm hmm) that they want, like they want to oversee everything and make sure it’s all done to like high level of care*” (Dietitian Assistant, Site D). Although these traits were reported to cause barriers to delegation, they did not apply universally to all dietitians; “*I could see that some dietitians might have some issue (mmm) in letting go…and then there would be others that are ‘yeah go for it’ (mmm) ‘please take it’ (yes), yeah and so then I guess it’s based on the personality of the dietitian*” (Management, Site D). These perceptions, however, were not reported by dietitian participants. Perceptions were mostly favourable towards dietitian assistants being willing and able to embrace change; however, it was acknowledged that some dietitian assistants may potentially struggle with delegation; “*In general everyone accepts it because it’s part of the job*” (Dietitian Assistant, Site C).

Personal attitudes and beliefs about delegation varied. Globally, participants appeared positive towards the concept of delegation, with 22 of 23 responding positively to the initial interview question, “What are your thoughts around delegating malnutrition care activities to assistants?”, indicating some optimistic attitudes. Managers and dietitian assistants were more positive and willing regarding delegation. However, exploration beyond these initial responses highlighted that managers and dietitian assistants generally appeared more likely to have positive attitudes towards delegation than the dietitian cohort. “*I wish I could wave a magic wand (yeah) and everyone get as excited about it as what [manager name] and I are*” (Food Service, Site A). Despite dietitians expressing optimistic attitudes, they were often perceived by others to have negative attitudes or beliefs around delegation; these ranged from fears around job security or performance to being overly safety-conscious. “*We’ve got a large temporary workforce, staffing workforce, so I think their fear that well* “*if we delegate to the nutrition assistants, are they going to think that they can do our job (mmm) and they don’t need us*” (Management, Site A). Dietitian assistant attitudes exhibited patient-centredness and desire to provide useful assistance to dietitians. “*Our basic thing is to make sure the patient gets the right care*” (Dietitian Assistant, Site C).

The final component of ‘human factors’ described by participants was focused on the need for trusting relationships for delegation to work, “*[the dietitian] needs to be able to, to just do that (mmm), delegate and, and trust that I’m going to go and do it*” (Dietitian Assistant, Site E/F). Positive relationships between the dietitian and dietitian assistant enabled delegation; “K*nowing that I can trust that it’ll [patient] be thrown back to me if they are needed, if I am needed (mmm) to see them [patient]*” (Dietitian, Site F). Poor relationships and distrust were consistently highlighted as a key barrier to delegation.

### 3.2. Balancing Value and Risk of Delegation

The perception of balancing value and risk associated with delegation of malnutrition care activities to dietitian assistants emerged as an important theme for delegation. Most participants, regardless of role, acknowledged the potential benefits of delegation for patients, interdisciplinary healthcare workers, clinical services, and funders. Perceived patient benefits included increased timeliness of care, a reduction in the proportion of patients at risk of malnutrition who were not receiving care, meeting service expansions and increasing demands, as well as improved care processes and patient engagement; “*Be able to get an established clinical role, supportive role from the DAs [assistants] umm I think is a, is a way forward for how the workforce is going to be able to cope with those increased demands*” (Management, Site D).

Potential benefits for dietitian assistants and dietitians were also observed, including increasing opportunities to work to full scope of practice, undertake quality assurance processes and freeing up time for research, “*We can obviously prioritise the higher sort of malnutrition risk cases, and then reinvest time into further research*” (Dietitian site C). A further potential benefit was improved workforce experiences and teamwork; “*It’s gonna really increase just our [assistants] job satisfaction, just us feeling like we’ve got a little bit more of a clinical role*” (Dietitian Assistant Site D). Improved value of healthcare provision, both in terms of what matters to consumers and the costs of service provision was another perceived benefit.

Despite these potential benefits to patients, staff and services, perceived risks were also reported, mostly by dietitian participants. These included: potential inappropriate clinical care or harm, poor documentation, missed diagnosis(es), funding and resourcing implications, and dietitian assistants working beyond the scope of practice; “*What I understand about documentation of malnutrition and then therefore the funding that we receive, that it’s something that the dietitians are still going to have to keep a handle on*” (Dietitian, Site F). Conversely, dietitian assistants appeared to be generally aware to not practice outside their scope and were patient-focused, ensuring the correct care was provided to the patient; “*You would have to be careful that it’s not too much and not too much into the role of the dietitian*” (Dietitian Assistant, Site A). Participants reflected that having procedures and risk management processes in place would be a key strategy to prevent harm; “*just to provide the safety net so that they [assistants] don’t get into any trouble*” (Dietitian, Site B).

### 3.3. Creating Competence, Capability and Capacity

The competence, capability, and capacity to delegate and receive delegation appeared to be dependent on effective communication, background and training, and clinical governance. Communication from the dietitian assistant to dietitian regarding concerns was reported to be essential; “*Just knowing that there’s that robust communication back to the dietitian when they’ve [dietitian assistant] implemented something for one of their patients*” (Food Service, Site D). Communication was also said to be crucial to dietitian assistants’ ability to undertake the delegated patient care or task. However, effectiveness of the communication between the dietitian and dietitian assistant appeared to vary from site to site and from person to person; “*Some [dietitian] are very clear and have all the information and yeah, you don’t need to ask any questions about what they’ve delegated (yeah) it’s just there, you have everything*” (Dietitian Assistant, Site B).

The background, training, and experiences of dietitian assistants appeared to affect their confidence and the confidence placed in them by dietitians; “*It is such a diverse workforce I think it is dependent on who you’re delegating to, how much confidence you have in their ability to complete the task*” (Dietitian, Site A). Whilst dietitian assistants with food service backgrounds were acknowledged for their knowledge of the local food service systems, this appeared to be viewed as a barrier for clinical delegation. More confidence appeared to be associated with dietitian assistants with additional qualifications, ranging from a certificate to dietitian degrees. “*Certificate that they’re [assistants] doing (oh yeah), but that’s probably actually going to help a lot in terms of their knowledge*” (Dietitian, Site C). Managers and dietitians who had long-standing experience with delegation appeared more positive than those newer to the profession or with limited or no delegation experience. “W*e’ve been doing it here for years and it’s worked really well*” (Food Service, Site B).

We identified diverse role opportunities to progress the dietitian assistant workforce to undertake activities across nutrition care processes (e.g., triaging referrals, collecting assessment and diagnostic data, food interventions, fluid and supplements, education, care coordination, monitoring, and clinical handover); “*There’s nothing that I [manager] can think of at the moment that (yep) that we shouldn’t be doing [delegating], other than umm commencement of enteral feeds*” (Management, Site D). Dietitian assistants were generally not always aware of what further tasks they should progress towards doing for malnourished patients, emphasizing the need for training; “*I’m not sure what other malnutrition stuff is out there that they can delegate for us, we don’t know any of that*” (Dietitian Assistant, Site C). Training was considered essential prior to implementation of delegation; “*Having everyone who’s going to be coming through the wards (mmm) is trained and is confident and competent (yep) to do it*” (Dietitian, Site A). Professional development opportunities for dietitian assistants could be used develop this capability and capacity. However, undertaking formal competency assessment of dietitian assistants for malnutrition care was viewed as both an enabler and barrier. Some participants felt this was helpful, whereas others believed that this was a time barrier and stalled progression of delegation; “*Even just the time to implement and umm do all the training and stuff for the frameworks*” (Management, Site B). There were varying views and practices regarding how training and supervision should happen; some were more reliant on team leaders and managers, and others on peer shadowing. “*Feedback (oh yep) and us [DAs] shadowing is always good*” (Dietitian Assistant, Site B).

Clinical governance processes, guidelines, and systems to ensure safe patient care and to support practicing within scope were perceived to increase the likelihood of successful delegation. “*Definitely things you can put in place, you know Calderdale framework, training or whatever it is (mmm hmm) to kind of make that all official and make sure that there’s appropriate safety nets*” (Management, Site F). Specific criteria and instructions regarding appropriate patients for delegation was desirable, with most participants suggesting that complex patients should stay under dietitian care. However, there were varying views expressed regarding what ‘complex’ might look like. Patient factors and the appropriateness to delegate particular patients was also discussed. Some patient groups were considered challenging to be delegated to dietitian assistants; “*I think it gets a bit hard if you’ve got dementia patients*” (Dietitian Assistant, Site E/F). Due to communication challenges with certain groups of patients, the ability to deliver all components of nutrition care may not be achievable, regardless of the treating dietitian’s or dietitian assistant’s qualification, competence, capability, or capacity. Others highlighted that it is difficult to develop delegation guidelines and instructions for populations with varying levels of cognitive impairment.

To promote capacity and capability, team leaders and managers believed that they needed to take the lead to influence delegation within their departments; “*Professional supervision, team leadership and talking about these things, on how often do you, how frequently do you delegate to the nutrition assistants, having a look at handover sheets and kind of talking through patients and you know being actively involved*” (Management, Site B). Executive-level leadership and union engagement were also considered to be essential for changing delegation practice.

### 3.4. Recognizing Contextual Factors

Participants described resourcing delegation, challenging workloads, and changing climates as factors that could affect implementation of delegation. Competing demands for resources and lack of budget were routinely considered a barrier, although what this looked like varied across sites. Not having a budget to fund dietitian assistant positions was an obvious impacting factor for delegation; “*That’s a fairly straight forward one, we don’t have any clinical assistants here*”. (Management, Site G). Other sites expressed difficulties regarding access to dietitian assistant staff backfill. “W*hen [assistant name] is away [laughter] (yep), we have to change the way the department works*” (Management, Site F). Participants highlighted the need to consider innovative ways to resource dietitian assistant positions within budgetary constraints, for example, through workforce redesign; “*Allied health then needs to have conversation around, are we prepared to give up a HP4 [health professional level 4] and turn it into 2 allied health assistants*” (Management, Site G). However, challenges with this approach were also acknowledged, for example, compliance with union and human resource requirements.

Challenging workloads were perceived to be both a barrier and an enabler of delegation. Dietitians were reported to be busy and routinely unable to action all referrals. Being unable to attend to referrals appeared to be a motivating factor to implement delegation for some, as they saw that delegation could decrease their workload; “*It’s [delegation] an ability for the dietitian to be able to offload some of their workload so that they can continue to stay afloat*” (Food Service, Site C). Conversely, other participants observed that they were too busy to implement delegation; “*Having time and capacity to train the NAs and develop the tools in the first place*” (Dietitian, Site A). A common workload barrier was that menu-related activities of the assistant role would limit their ability to undertake malnutrition care activities. For example, “*We need a certain number of umm feet on the ground in order to collect the menus across the hospital*” (Food Service, Site C). Some sites reported creative ways to incorporate both menu duties and malnutrition care activities within a dietitian assistant role. Whilst some participants expressed concerns associated with re-evaluation of roles, others observed that role and scheduling changes would have a positive impact on delegation as more time and opportunity could be available in the dietitian assistant workload; “*She [assistant] would have less of a task of doing the malnutrition screening in the background, therefore she’d have more time to be delegated things (mmm), and could spend more time with patients…*” (Dietitian, Site F).

Evolving, broad influencing factors perceived to impact delegation to dietitian assistants were framed as the changing care climate. These factors included the pressure and burden of ever-growing service expectations; requirements in the absence of additional resources; equity of access; availability; (dis)engagement of interdisciplinary healthcare workers; and responsiveness to physical, cultural, and social complexities; “*With healthcare services increasing and umm funding not being equivalent (yep) we need to find new models of care to be able to deliver high quality services*” (Management, Site B). While generally considered an enabler of delegation, policy, governance, and professional guidelines were observed to also be potential barriers to expansion of dietitian assistant roles. For example, with regards to delegating subjective global assessments to dietitian assistants, a manager reported, “*I was just reading the DAA [Dietitians Association of Australia] position paper actually, because I was on the DAA site for something else, and I wondered if I needed to revise my position after reading that*” (Management, Site F). Whether academic and clinical education sectors are keeping pace with changing healthcare climates was also questioned, with some participants unsure whether delegation models or anything regarding the dietitian assistant role is taught to pre-graduates. It was evident through the interviews that there was considerable uncertainty regarding acceptance of delegation practices by new graduate dietitians; “*I think a lot of new graduate dietitians might be hesitant in completely giving over care to an NA [nutrition assistant]*” (Dietitian, Site B).

## 4. Discussion

This is the first qualitative study to explore barriers and enablers to delegation of malnutrition care activities to dietitian assistants within the hospital setting. Our findings demonstrate that working with the human factors; balancing value and risk of delegation; creating competence, capability, and capacity; and recognizing contextual factors are key influencers of successful delegation of malnutrition care to dietitian assistants.

The views, perceptions, and opinions of staff members regarding delegation to dietitian assistants influenced why ‘below-scope’ tasks are still being retained by dietitians rather than being delegated to dietitian assistants where possible and safe to do so [8,19]. Commonly perceived humanistic traits of clinical dietitians were to fear harm, be risk-averse, resist change, and distrust the delegation process and capacity of dietitian assistants. Prior work suggests that public health registered dietitians in the UK were less likely to be early adopters, and terms including “less flexible”, “conservative”, “fear”, and “reluctance” were used when referring to the wider profession [26]. Carefully unpacking and addressing these human factors at site, professional, and individual levels is an urgent priority if full-scope delegation-of-care actions are taken up beyond ‘early adopter’ clinical dietetics services [27].

It is becoming increasingly clear that dietitian assistant roles can positively influence patient, healthcare, and workforce outcomes, especially when used within the context of a multidisciplinary team [18,28,29]. Additionally, the dietetic workforce has identified and prioritised delegation of malnutrition care as an opportunity to facilitate high-value healthcare [20]. However, malnutrition care appears to still predominately be undertaken by dietitians [8,14,19]. This introduces another novel insight: the cognitive dissonance observed in relation to delegation to dietitian assistants. Cognitive dissonance is the conflict experienced when behaviour contradicts one’s beliefs and, in the medical setting, is known to influence decision making and can lead to difficulty in changing practices [30,31]. This study has shown that although clinical dietitians and service managers report valuing delegation, failure to delegate was justified by lack of time, resources, models of care, and access to appropriate staff. This study further confirms previous quantitative findings that suggested a gap between a belief or feeling that delegation is a good idea and the action of actually delegating care [19].

A prior superficial assessment suggested that managers and dietitian assistants mostly seemed ready to support delegation [19]. However, results from this study highlight a lack of access to dietitian assistant staff as a key barrier to delegation. This is not surprising, given that even where available, the dietitian assistant workforce is much smaller than that of their dietitian counterparts [32,33]. Our exploratory work here does raise questions regarding whether managers and their executives adequately value delegation through prioritisation of funding to create and maintain dietitian assistant roles. Whether there is adequate leadership, engagement, and influence with professional associations and the academic sector to ensure governance processes and activities to improve competence, capability, and capacity must also be considered to support delegation processes that are evolving with the rapidly changing healthcare climate.

Our results highlight that unless there is a more consistent level of competence and capability developed within the dietitian assistant workforce, as well as supportive contextual factors/systems and resources to support delegation, the capacity for clinical dietitians to trust ‘full-scope’ dietitian assistants with malnutrition care tasks may be undermined. Results also suggest drawing some lines of distinction between dietitian assistant versus dietitian scope of practice, for example, prescribing enteral or parenteral nutrition, would be helpful to build this trust and align with existing governance documents [34,35]. Data suggest that dietitian assistants’ qualifications influenced dietitian confidence and trust. It appears that dietitian assistants with higher qualifications, such as university degrees or certificates, are more trusted than those without qualifications or from food service roles. Although there are existing resources, such as Allied Health Assistant Frameworks, statewide clinical task instructions (including competencies) and scope documents, the ‘competence, capability, and capacity’ of staff surrounding delegation, at face value, appears to still contribute to barriers to progressing delegation [34,35,36,37,38].

Our findings suggest further exploration and explanation is required regarding the apparent cognitive dissonance found with dietitians and unpacking the black box between intention and actual behaviour change [39]. Implementation theory recognizes that successful behaviour change requires moving beyond simplistic solutions that target capability and opportunities to those that focus on the automatic and reflective processes that motivate change, ranging from social and professional roles and identities to beliefs about capabilities, consequences, emotions, intentions, and optimism [40].

Whilst evidence regarding allied health assistant contributions more broadly is limited, research has shown that there may be patient and organisational benefits of delegation of therapy to a range of allied health assistants [41]. Our results reiterate that where not already embedded, delegation of malnutrition care activities to dietitian assistants will require attention to the process of implementation. Teams must work together to identify and implement improved delegation activities and apply locally tailorable models, together with theories, models, and/or frameworks that have proven successful in other settings and are considerate of local contexts [8,22,42,43,44,45]. Iterative, data-informed action cycles are needed to move towards delegation. These cycles would also be supported by developing clinical governance, undertaking education, and creating resources to support delegation processes. For example, statewide clinical task instructions, statewide allied health assistant frameworks, and local workplace instructions that can be shared amongst sites to positively influence the barriers and enablers identified in this study.

### Strengths and Limitations

There was even representation across dietitians, dietitian assistants, and managers, and some representation of food service managers. There was also diversity in the sample based on dietetic department size, dietitian assistant staffing, delegated models of care, facility size, and patient populations, providing the opportunity to capture a broad range of experiences (Appendix A). The nature of individual interviews allowed for participants to discuss topics, ideas, and feelings and provided a safe space to disclose information. The limitations of this study were that interviews and initial analysis were undertaken by an investigator (AR) whose usual role is as a dietitian assistant and who had built relationships with some participants through their involvement in the malnutrition care research program (SIMPLE II). Although this potential bias and conflict were declared at the beginning of interviews, this influenced the content of interviews, as well as the analytical stance. Members of the authorship team cover a breadth of experience and skills, and their validation of themes mitigated this bias.

## 5. Conclusions

This study highlights novel insights into barriers and enablers to delegating malnutrition care to dietitian assistants. Appreciating the unique perspectives of humans as individuals and in their collective healthcare roles; moving from words to actions that value delegation; engaging in processes to improve the competency, capability, and capacity of all players; and being responsive to climate and contextual factors were identified as key determinants of successful delegation of malnutrition care to dietitian assistants.

## Figures and Tables

**Table 1:** Elements used for purposive sampling of participants.

Element	Purposive Sampling
Position	Dietetics director/lead Food services manager or food services dietitian Clinical dietitian Dietitian assistant with qualifications (e.g., degree or certificate) Dietitian assistant with food services background *
Site	Hospital size (beds) and locality (metropolitan, regional, rural/remote)
Experience	Years of experience and workload (full time, part time, casual)
Resources	Access to assistant staff, food services models, funded dietetics full-time equivalent positions per 100 beds

* Dietitian assistants who have been trained as and worked as food service workers prior to working as a dietitian assistant.

**Table 2:** Demographic details of interview participants.

Demographic	Dietitian Assistant *n*, (%)	Dietitian *n*, (%)	Management * n, (%)	Food Services ** n, (%)
Sex				
Female	7 (100)	5 (83)	5 (83)	4 (100)
Male	0 (0)	1 (17)	1 (17)	0 (0)
Employment Type				
Full time	2 (29)	5 (83)	5 (83)	4 (100)
Part time	5 (71)	1 (17)	1 (17)	0 (0)
Years practicing				
2–5	4 (57)	3 (50)	0 (0)	0 (0)
6–10	0 (0)	2 (33)	1 (17)	0 (0)
11–20	2 (29)	1 (17)	3 (50)	4 (100)
21–30	1 (14)	0 (0)	2 (33)	0 (0)
**Total 23**	**7 (31)**	**6 (26)**	**6 (26)**	**4 (17)**

* Management includes directors of dietetics and team leaders in dietetics. ** Food services includes food service dietitians, team leaders in food services and dietetics, and food service managers.

## Data Availability

Please contact the corresponding author regarding data availability.

## Appendix A

**Table A1:** Semi-structured interview guide.

Semi-Structured Interview Questions Guide
What are your thoughts around delegating malnutrition care activities to assistants?
Do you think assistants are used enough for malnutrition care activities? What are some of the reasons why we might not delegate? What are the things that would help more delegation?
Do you think delegating malnutrition care activities is a good idea in principle; why/why not?
Tell me about when and where you think delegation of malnutrition care activities might fail in the real world? Why do you think that?
What do you think are the challenges to delegating malnutrition care activities to assistants?
Is there anything that you think can or could facilitate or enable successful delegation of malnutrition care? Why do you think this would work?
What do you think might influence confidence or capability of an **assistant** to perform a delegated malnutrition care activity?
What do you think might influence confidence or capability of a **dietitian** to delegate malnutrition care activities to assistants?
What malnutrition care activities do you think should / shouldn’t be delegated to assistants—why/why not?
For assistants: Do you think dietitians do a good job at delegating? Why / why not? Do you think some dietitians are better at delegating than others? What are the things you think about when deciding if they did a good job at delegating?
For dietitians: What things influence your decision to delegate malnutrition care activities to assistants? Is your decision influenced by who you are delegating to? If so, what are the factors that influence this decision
If you could change one thing about delegation to assistants what would it be? Why?

Closing: Is there anything else you’d like to say that you haven’t had the chance to yet? Thank you.

## Appendix B

**Table A2:** Participant site demographics.

Site	Bed Numbers	Metro/Regional	Number of Dietitians	Number of Dietitian Assistants
A	450–599	Tertiary and satellite centres	44	14
B	600–749	Tertiary	20	5
C	150–299	Regional	8	6
D	450–599	Tertiary	26	10
E	0–149	Regional	1	1 *
F	150–299	Regional	6	1 *
G	300–449	Regional	6	0

* A single dietitian assistant worked across these two sites

## References

[1] Cederholm T., Jensen G.L., Correia M.I.T.D., Gonzalez M.C., Fukushima R., Higashiguchi T., Baptista G., Barazzoni R., Blaauw R., Coats A.J. (2019). GLIM criteria for the diagnosis of malnutrition—A consensus report from the global clinical nutrition community. J. Cachexia Sarcopenia Muscle.

[2] Adams N.E., Bowie A.J., Simmance N., Murray M., Crowe T.C. (2008). Recognition by medical and nursing professionals of malnutrition and risk of malnutrition in elderly hospitalised patients. Nutr. Diet..

[3] Agarwal E., Ferguson M., Banks M., Bauer J., Capra S., Isenring E. (2012). Nutritional status and dietary intake of acute care patients: Results from the Nutrition Care Day Survey 2010. Clin. Nutr..

[4] Barker L.A., Gout B.S., Crowe T.C. (2011). Hospital malnutrition: Prevalence, identification and impact on patients and the healthcare system. Int. J. Environ. Res. Public Health.

[5] Isabel T.D., Correia M., Waitzberg D.L. (2003). The impact of malnutrition on morbidity, mortality, length of hospital stay and costs evaluated through a multivariate model analysis. Clin. Nutr..

[6] Lim S.L., Ong K.C.B., Chan Y.H., Loke W.C., Ferguson M., Daniels L. (2012). Malnutrition and its impact on cost of hospitalization, length of stay, readmission and 3-year mortality. Clin. Nutr..

[7] Bell J.J., Bauer J., Capra S., Pulle R.C. (2014). Quick and Easy Is Not without Cost: Implications of Poorly Performing Nutrition Screening Tools in Hip Fracture. J. Am. Geriatr. Soc..

[8] Bell J.J., Young A., Hill J., Banks M., Comans T., Barnes R., Keller H.H. (2018). Rationale and developmental methodology for the SIMPLE approach: A Systematised, Interdisciplinary Malnutrition Pathway for impLementation and Evaluation in hospitals. Nutr. Diet..

[9] Keller H., Laur C., Valaitis R., Bell J., McNicholl T., Ray S., Murphy J., Barnes S., for the More-2-Eat team (2017). More-2-Eat: Evaluation protocol of a multi-site implementation of the Integrated Nutrition Pathway for Acute Care. BMC Nutr..

[10] McCamley J., Vivanti A., Edirippulige S. (2019). Dietetics in the digital age: The impact of an electronic medical record on a tertiary hospital dietetic department. Nutr. Diet..

[11] Australian Commission on Safety and Quality in Health Care (2021). National Safety and Quality Health Service Standards. Comprehensive Care Standard.

[12] (2017). Queensland Health, Framework for Effective and Efficient Dietetic Services: Malnutrition.

[13] Ferguson M., Banks M., Bauer J., Isenring E., Vivanti A., Capra S. (2010). Nutrition screening practices in Australian healthcare facilities: A decade later. Nutr. Diet..

[14] Bell J.J., Young A.M., Hill J.M., Banks M.D., Comans T.A., Barnes R., Keller H.H. (2021). Systematised, Interdisciplinary Malnutrition Program for impLementation and Evaluation delivers improved hospital nutrition care processes and patient reported experiences—An implementation study. Nutr. Diet..

[15] Value-Based Healthcare—Shifting from Volume to Value.

[16] Tappenden K.A., Quatrara B., Parkhurst M.L., Malone A.M., Fanjiang G., Ziegler T.R. (2013). Critical role of nutrition in improving quality of care: An interdisciplinary call to action to address adult hospital malnutrition. JPEN J. Parenter. Enteral. Nutr..

[17] Keller H., Koechl J.M., Laur C., Chen H., Curtis L., Dubin J.A., Gramlich L., Ray S., Valaitis R., Yang Y. (2020). More-2-Eat implementation demonstrates that screening, assessment and treatment of malnourished patients can be spread and sustained in acute care; a multi-site, pretest post-test time series study. Clin. Nutr..

[18] Rushton A., Edwards A., Bauer J., Bell J.J. (2021). Dietitian assistant opportunities within the nutrition care process for patients with or at risk of malnutrition: A systematic review. Nutr. Diet..

[19] Rushton A., Young A., Keller H., Bauer J., Bell J. (2021). Delegation Opportunities for Malnutrition Care Activities to Dietitian Assistants—Findings of a Multi-Site Survey. Healthcare.

[20] Rushton A., Elmas K., Bauer J., Bell J. (2021). Identifying Low Value Malnutrition Care Activities for De-Implementation and Systematised, Interdisciplinary Alternatives—A Multi-Site, Nominal Group Technique Approach. Nutrients.

[21] Michie S., Van Stralen M.M., West R. (2011). The behaviour change wheel: A new method for characterising and designing behaviour change interventions. Implement. Sci..

[22] Damschroder L.J., Aron D.C., Keith R.E., Kirsh S.R., Alexander J.A., Lowery J.C. (2009). Fostering implementation of health services research findings into practice: A consolidated framework for advancing implementation science. Implement. Sci..

[23] Laur C., Valaitis R., Bell J., Keller H. (2017). Changing nutrition care practices in hospital: A thematic analysis of hospital staff perspectives. BMC Health Serv. Res..

[24] Virginia Braun V.C. (2013). Successful Qualitative Research: A Practical Guide for Beginners.

[25] Saldana J. (2016). The Coding Manual for Qualitative Researchers.

[26] Abrahams M., Frewer L.J., Bryant E., Stewart-Knox B. (2018). Perceptions and experiences of early-adopting registered dietitians in integrating nutrigenomics into practice. Br. Food J..

[27] Miake-Lye I., Mak S., Lam C.A., Lambert-Kerzner A.C., Delevan D., Olmos-Ochoa T., Shekelle P. (2020). Scaling Beyond Early Adopters: A Content Analysis of Literature and Key Informant Perspectives. J. Gen. Intern. Med..

[28] Bell J.J., Bauer J.D., Capra S., Pulle R.C. (2014). Multidisciplinary, multi-modal nutritional care in acute hip fracture inpatients—Results of a pragmatic intervention. Clin. Nutr..

[29] Young A.M., Banks M.D., Mudge A. (2018). Improving nutrition care and intake for older hospital patients through system-level dietary and mealtime interventions. Clin. Nutr. ESPEN.

[30] Festinger L. (1957). A Theory of Cognitive Dissonance.

[31] Klein J., McColl G. (2019). Cognitive dissonance: How self-protective distortions can undermine clinical judgement. Med. Educ..

[32] (2015). Urbis, Dietitian and Nutrition Assistant Workforce Mapping. https://www.health.nsw.gov.au/workforce/alliedhealth/Documents/dietetic-assistant-support-worker.pdf.

[33] Allied Health Professions’ Office of Queensland Allied Health Workforce Indicators 2013–2018.

[34] Allied Health Professions’ Office of Queensland (2015). Allied Health Assistant Framework.

[35] Andersen D., Baird S., Bates T., Chapel D.L., Cline A.D., Ganesh S.N., Garner M., Grant B.L., Hamilton K.K., Jablonski K. (2017). Academy of Nutrition and Dietetics: Revised 2017 Scope of Practice for the Nutrition and Dietetics Technician, Registered. J. Acad. Nutr. Diet..

[36] Dietitians Association of Australia (2016). Scope of Practice-Support Staff in Nutrition and Dietetic Services.

[37] Queensland Health (2021). Clinical Task Instructions. https://www.health.qld.gov.au/ahwac/html/clintaskinstructions.

[38] (2010). The British Dietetic Association, Dietetic Support Worker & Assistant Practitioner Roles. https://www.bda.uk.com/publications/apdswcareer.

[39] Grimshaw J.M., Presseau J., Tetroe J., Eccles M.P., Francis J.J., Godin G., Graham I.D., Hux J.E., Johnston M., Légaré F. (2014). Looking inside the black box: Results of a theory-based process evaluation exploring the results of a randomized controlled trial of printed educational messages to increase primary care physicians’ diabetic retinopathy referrals [Trial registration number ISRCTN72772651]. Implement. Sci..

[40] Atkins L., Francis J., Islam R., O’Connor D., Patey A., Ivers N., Foy R., Duncan E., Colquhoun H., Grimshaw J.M. (2017). A guide to using the Theoretical Domains Framework of behaviour change to investigate implementation problems. Implement. Sci..

[41] Snowdon D.A., Storr B., Davis A., Taylor N.F., Williams C.M. (2020). The effect of delegation of therapy to allied health assistants on patient and organisational outcomes: A systematic review and meta-analysis. BMC Health Serv. Res..

[42] Cane J., O’Connor D., Michie S. (2012). Validation of the theoretical domains framework for use in behaviour change and implementation research. Implement. Sci..

[43] Graham I.D., Logan J., Harrison M.B., Straus S.E., Tetroe J., Caswell W., Robinson N. (2006). Lost in Knowledge Translation: Time for a Map?. J. Contin. Educ. Health Prof..

[44] Bell J.J., Rossi T., Bauer J.D., Capra S. (2014). Developing and evaluating interventions that are applicable and relevant to inpatients and those who care for them; a multiphase, pragmatic action research approach. BMC Med. Res. Methodol..

[45] Passfield J., Nielsen I., Brebner N., Johnstone C. (2018). Skill sharing and delegation practice in two Queensland regional allied health cancer care services: A comparison of tasks. Aust. Health Rev..

